# Physiological responses to cuddling babies with hypoxic–ischaemic encephalopathy during therapeutic hypothermia: an observational study

**DOI:** 10.1136/bmjpo-2021-001280

**Published:** 2021-12-16

**Authors:** David Odd, Satomi Okano, Jenny Ingram, Peter S Blair, Amiel Billietop, Peter J Fleming, Marianne Thoresen, Ela Chakkarapani

**Affiliations:** 1 Population Medicine, Cardiff University, School of Medicine, Cardiff, UK; 2 Neonatology, St Michael's Hospital, University Hospitals Bristol and Weston NHS Foundation Trust, Bristol, UK; 3 Translational Health Sciences, University of Bristol Medical School, Bristol, UK; 4 Centre for Academic Child Health, University of Bristol Medical School, Bristol, UK; 5 Centre for Child and Adolescent Health, University of Bristol Medical School, Bristol, UK; 6 Neonatal Intensive Care Unit, North Bristol NHS Trust, Westbury on Trym, Bristol, UK

**Keywords:** neonatology, physiology

## Abstract

**Objectives:**

To determine whether parents cuddling infants during therapeutic hypothermia (TH) would affect cooling therapy, cardiorespiratory or neurophysiological measures. The secondary aim was to explore parent–infant bonding, maternal postnatal depression and breastfeeding.

**Design:**

Prospective observational study.

**Setting:**

Two tertiary neonatal intensive care units (NICU).

**Participants:**

Parents and their term-born infants (n=27) receiving TH and intensive care for neonatal hypoxic–ischaemic encephalopathy.

**Interventions:**

Cuddling up to 2 hours during TH using a standard operating procedure developed in the study (CoolCuddle).

**Main outcome measures:**

Mean difference in temperature, cardiorespiratory and neurophysiological variables before, during and after the cuddle. Secondary outcomes were parental bonding, maternal postnatal depression and breastfeeding.

**Results:**

During 70 CoolCuddles (115 cumulative hours), there were measurable increases in rectal temperature (0.07°C (0.03 to 0.10)) and upper margin of amplitude-integrated electroencephalogram (1.80 µV (0.83 to 2.72)) and decreases in oxygen saturations (−0.57% (−1.08 to −0.05)) compared with the precuddle period. After the cuddle, there was an increase in end-tidal CO_2_ (0.25 kPa (95% CI 0.14 to 0.35)) and mean blood pressure (4.09 mm Hg (95% CI 0.96 to 7.21)) compared with the precuddle period. From discharge to 8 weeks postpartum, maternal postnatal depression declined (13 (56.5%) vs 5 (23.8%), p=0.007); breastfeeding rate differed (71% vs 50%, p=0.043), but was higher than national average at discharge (70% vs 54.6%) and mother–infant bonding (median (IQR): 3 (0–6) vs 3 (1–4)) remained stable.

**Conclusion:**

In this small study, CoolCuddle was associated with clinically non-significant, but measurable, changes in temperature, cardiorespiration and neurophysiology. No infant met the criteria to stop the cuddles or had any predefined adverse events. CoolCuddle may improve breastfeeding and requires investigation in different NICU settings.

**What is known about the subject?**

- Infants undergoing therapeutic hypothermia for neonatal hypoxic–ischaemic encephalopathy have improved motor outcomes, but still have a greater risk of cognitive impairments.
- Lack of physical and emotional parent–infant interaction during therapeutic hypothermia and intensive care may adversely affect parent–infant bonding impacting children’s cognitive development.
- Data promoting parents cuddling their babies during cooling are sparse.

**What this study adds?**

- Using CoolCuddle, the target temperature of cooling therapy, ventilation, cardiorespiratory and neurophysiological measures remained within the normal ranges.
- Measurable differences in ventilation, electroencephalogram voltages, core temperature and blood pressure were identified; although none appeared clinically important.
- Between the 1-week and 8-week time points, mother–infant bonding measures were similar and postnatal depression scores improved. Breastfeeding was higher than the national average.

## Introduction

Therapeutic hypothermia (TH), cooling the core temperature to 33.5°C, along with intensive care is the standard treatment for babies with hypoxic–ischaemic encephalopathy (HIE) following birth asphyxia.^1^ While TH has significantly reduced the incidence^2 3^ of cerebral palsy (CP) in survivors of HIE, those without CP still have lower cognitive scores compared with their peers^4^ associated with disrupted brain structural connectivity.^5^

Children’s cognition might be improved by facilitating parent infant bonding,^6^ which is adversely affected by TH^7^ exacerbated by the inability of parents to interact physically with their baby during cooling therapy.^8^ Additionally, the high-tech intensive care environment and early physical separation soon after birth necessitated by the requirement for urgent cooling therapy and intensive care^9^ impairs bonding and parental mood.^10^ Almost 96% of surveyed neonatal units in the UK do not support parents cuddling their babies during cooling therapy due to concerns of temperature instability during cooling therapy and the impact on delivering intensive care.^11^

There is an urgent need to develop interventions to enable parents to interact safely with their babies during cooling therapy. We aimed to investigate whether temperature during TH, intensive care and neurophysiology would be affected by parents cuddling babies receiving TH and intensive care, soon after commencing TH for up to 2 hours, during cooling or rewarming. We also evaluated parent–infant bonding, postnatal depression and breastfeeding rates.

## Methods

This prospective observational study was conducted at two tertiary neonatal intensive care units (NICU) between October 2019 and November 2020.

### Participants

Parents were eligible if their infants were born at ≥36 weeks gestation undergoing TH using a servo-controlled cooling device and intensive care for HIE.^12 13^ We excluded infants who received considerable cardiorespiratory support (one or more of: high-frequency oscillation, mean airway pressure >12 cmH_2_O, inhaled nitric oxide for persistent pulmonary hypertension, oxygen requirement >70%, more than one chest drain or ≥3 inotropes). Infants who had congenital anomalies or status epilepticus at the time of the cuddle as well as families who lacked English proficiency to complete questionnaires were also excluded. The protocol and the statistical analysis plan are available online (https://doi.org/10.5523/bris.3vs3y2wa9t4if2jo97ni4x4cae).^14^ Only one parent cuddled the baby during each cuddle, although the other parent may have been sitting by their side.

### Patient and public involvement

Parents of children who underwent TH before the CoolCuddle study informed us that the lack of physical and emotional interaction during cooling therapy and intensive care affected bonding with their babies. They preferred to have physical contact with their babies during cooling therapy. However, they were concerned whether cuddling their babies during cooling would affect their babies’ treatment. We had a parent advisory group comprising parents of infants who were cooled for HIE. They were involved in the design of the study and helped us in developing patient relevant study outcomes. They supported us with changes in the study design, including suggestions of offering cuddles any time before the end of cooling treatment to boost the recruitment during COVID-19 visiting restrictions. Parent advisory group members participated in the study steering meetings to support the conduct of the study. The plain English summary of the study results will be disseminated to the study participants.

### Study procedures

We refined the existing process for cuddling infants receiving intensive care using an iterative process to develop a standard operating procedure (SOP) involving parents and nurses to enable parents to cuddle their infants during TH (CoolCuddle). We administered CoolCuddle involving two nurses supervised by an advanced neonatal nurse practitioner online supplemental figure 1. Routine intensive care monitoring including single-channel amplitude-integrated electroencephalogram (aEEG) and regional cerebral oxygenation monitoring (rScO_2_) continued during the cuddle. Babies received morphine or fentanyl infusion during TH.^15^ We collected core and surface temperature, cardiorespiratory and neurophysiological data every 5 min for 1 hour during the precuddle and postcuddle, and for up to 2 hours during the cuddle epochs. For babies without continuous invasive blood pressure (BP) monitoring, non-invasive BP was measured every 15 min.

Cardiorespiratory data included heart rate, mean arterial BP, ventilatory parameters, peripheral oxygen saturation (SaO_2_) and blood gases (online supplemental table 1). Analgesic and inotropic support doses were collected. Pain was scored during the precuddle, cuddle and postcuddle using Neonatal Pain Agitation and Sedation Scale (N-PASS).^16^ We collected data on adverse events including accidental extubation, dislodgement of vascular catheters or aEEG electrodes and any incidence of needle-stick injury from EEG electrodes.

10.1136/bmjpo-2021-001280.supp3 Supplementary data

We assessed maternal postnatal depression using the Edinburgh Postnatal Depression Scale (EPDS),^17^ and maternal–infant bonding using the Mother–Infant Bonding Scale (MIBS)^18^ at 5–7 days and 8 weeks postpartum. Fathers’ attachment with their infants was assessed using the Paternal Postnatal Attachment Scale (PPAS)^19^ at 8 weeks postpartum. EPDS ≥13 was defined as indicative of depression.^20^ Breastfeeding rates at 1 and 8 weeks postpartum were collected.

### Outcomes

The primary outcome was physiological stability, defined as degree of variation in the rectal temperature, mean airway pressure, end-tidal CO_2_, fraction of inspired oxygen, heart rate, mean arterial BP, regional cerebral oxygenation and voltage of upper and lower margin of aEEG between the precuddle, cuddle and postcuddle epoch during the active cooling phase of TH. Secondary outcomes were breastfeeding rates, EPDS and MIBS scores at 5–7 days and 8 weeks postpartum, the proportion of mothers with EPDS ≥13^20^ or fathers with paternal postnatal attachment scores below the 25th and above the 75th percentile.^19^ A number of a-priori criteria of adverse effects on TH or intensive care were established to stop the cuddles (online supplemental table 2)

Sample size was chosen opportunistically and pragmatically based on a conservative estimate of the number of parents likely to consent to CoolCuddle within the constraints of time and staff availability to give precision to estimates but was not aimed at measuring efficacy or detecting differences in any individual physiological measure. In 2019, there were 62 eligible infants, we proposed a sample of minimum 24 to a maximum of 30 infants (48% recruitment rate).

### Statistical analysis

The main analysis data set was based on cuddles performed while TH was being delivered. A subset of additional cuddles occurred while rewarming took place. For those cuddles performed during active cooling (the primary cohort), the summary values for cardiorespiratory, temperature and neurophysiology measures for the hour before the cuddle, the period of cuddling and the hour after the cuddle, were summarised and compared between the three epochs. A multilevel, clustered linear model for the continuous measures (with the infant being the highest level, and then cuddle) was derived; with the likelihood ratio test used to assess if there was evidence of a difference between the three periods (the primary analysis) and absolute difference in measures (with 95% CIs) compared with the precuddle period derived. A logistic model with the same structure was then derived for binary measures (sleep–wake cycling, a high aEEG score) (online supplemental table 1). A sensitivity analysis was performed repeating the main analyses when CoolCuddles occurred during the rewarming phase of TH. In a post-hoc sensitivity analysis, we tested to see if the profile of any of the six main outcomes (MAP, mean BP, SpO_2_, EtCO_2_, heart rate and rectal temperature) varied by the number of cuddle (eg, first, second, etc.), the parent cuddling, the grade of HIE or if the cuddle was performed in the rewarming period).

Summary measures of maternal postnatal depression, mother–infant bonding and paternal postnatal attachment scores were derived, and paired measures were analysed using the Wilcoxon signed-rank test or McNemar’s test as appropriate. Results are presented as arithmetic mean (SD), geometric mean or mean change (95% CI) or number (%) as appropriate. Analysis was performed in Stata V.16.

## Results

**Figure 1:**
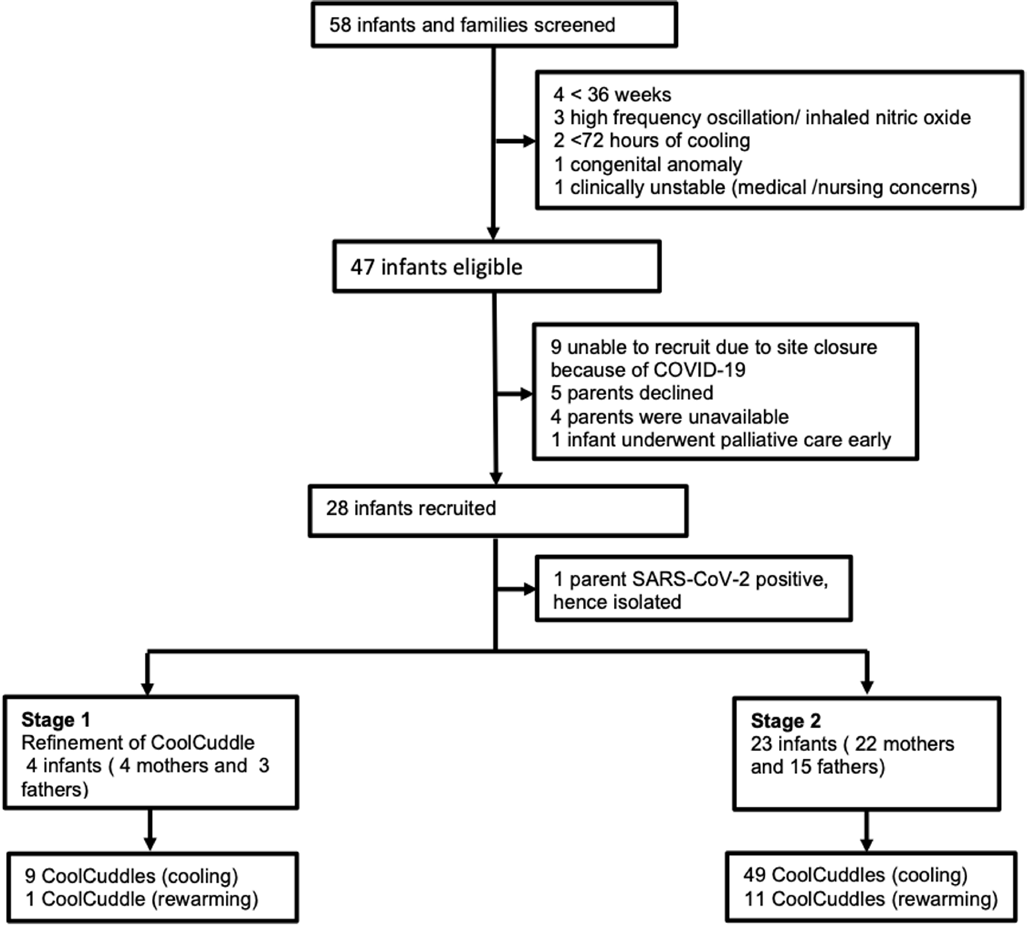
Study flow chart.

From 1 October 2019 to 30 November 2020, 58 infants received TH for HIE of which 27 infants were recruited (figure 1). Seventy CoolCuddles (12 during rewarming) were administered over a cumulative duration of 115 hours (18 hours during rewarming). Six children were cooled at one centre (Southmead) and 21 at the other (St Michael’s). The mean age for the first cuddle was at 50 hours (n=27), the second at 62 hours (n=22), the third at 70 hours (n=15) and the fourth at 74 hours (n=6) (online supplemental table 3). Demographics of the mothers, fathers and infants are shown in table 1. A total of 24 (17.8%) of cuddles were performed by the father. Babies received morphine infusion at a mean dose of 26.7 µg/kg/h (11.58) in 57 CoolCuddles, and fentanyl at 2.7 µg/kg/h (1.29) during 10 CoolCuddles; 25/27 (92.5%) babies were mechanically ventilated; 20/27 (74.1%) babies had central arterial lines. Babies received dopamine during 18/58 (31%) CoolCuddles. aEEG measures were not available for two infants.

**Table 1:** Characteristics of the study population

Measure	N*	Total
Maternal characteristics		
Age (years)	27	31.0 (4.9)
Race: White	27	24 (88.9%)
University qualification	26	13 (50.0%)
Pregnancy characteristics	27	
Primiparous		17 (63.0%)
Induction of labour		3 (11.1%)
Pregnancy complications†		5 (14.8%)
Intrapartum complications‡		23 (85.2%)
Lower segment caesarean section (LSCS)		11 (40.7%)
Breech		2 (7.4%)
Pyrexia >38°C, n (%)		0 (0.0%)
Paternal characteristics		
Age (years)	26	39.5 (1.6)
Race: White	25	21 (84.0%)
University qualification	20	9 (45.0%)
Infant characteristics	27	
Sex (male)		19 (70.4%)
Gestation weeks (mean (SD))		39.5 (1.5)
Birth weight g, mean (SD)		3314 (468)
Head circumference cm, mean (SD)		34.8 (1.3)
Transferred from LNU or SCBU for cooling		17 (63.0%)
Cord blood gas		
pH, mean (SD)	22	6.99 (0.16)
Base excess	21	−13.0 (6.0)
Apgar scores	27	
1 min		3 (1–5)
5 min		5 (4–7)
10 min		7 (4–8)
Need for respiratory support >10 min	27	22 (81.5%)
HIE grade	27	
I		3 (11.1%)
II		15 (55.6%)
III		9 (33.3%)
aEEG abnormality before TH	27	
Normal		2 (7.4%)
Moderately abnormal		21 (77.8%)
Severely abnormal		4 (14.8%)
Cardiac compressions		7 (25.9%)
Resuscitation drugs		1 (3.7%)
Age active cooling commenced (hours)	27	2.8 (1.3, 3.8)
Temperature at start of active cooling (°C)	26	34.8 (1.18)
Age when reached (hours) 33.5°C	27	5.3 (4.2)

Values are n (%), mean (SD) or median (IQR) as appropriate.

*N denotes number of subjects for whom data were available.

†Pre-eclampsia, HELLP (haemolysis, elevated liver enzymes and a low platelet count) syndrome, pregnancy-induced hypertension, antepartum bleed, diabetes, Bell’s palsy and polyhydramnios.

‡Cord prolapse, uterine rupture, shoulder dystocia, placental abruption, fetal decelerations, fetal bradycardia, prolonged rupture of membranes, reduced fetal movements, meconium-stained liquor.

aEEG, amplitude-integrated electroencephalogram; HIE, hypoxic–ischaemic encephalopathy; LNU, local neonatal unit; SCBU, special care baby unit; TH, therapeutic hypothermia.

**Figure 2:**
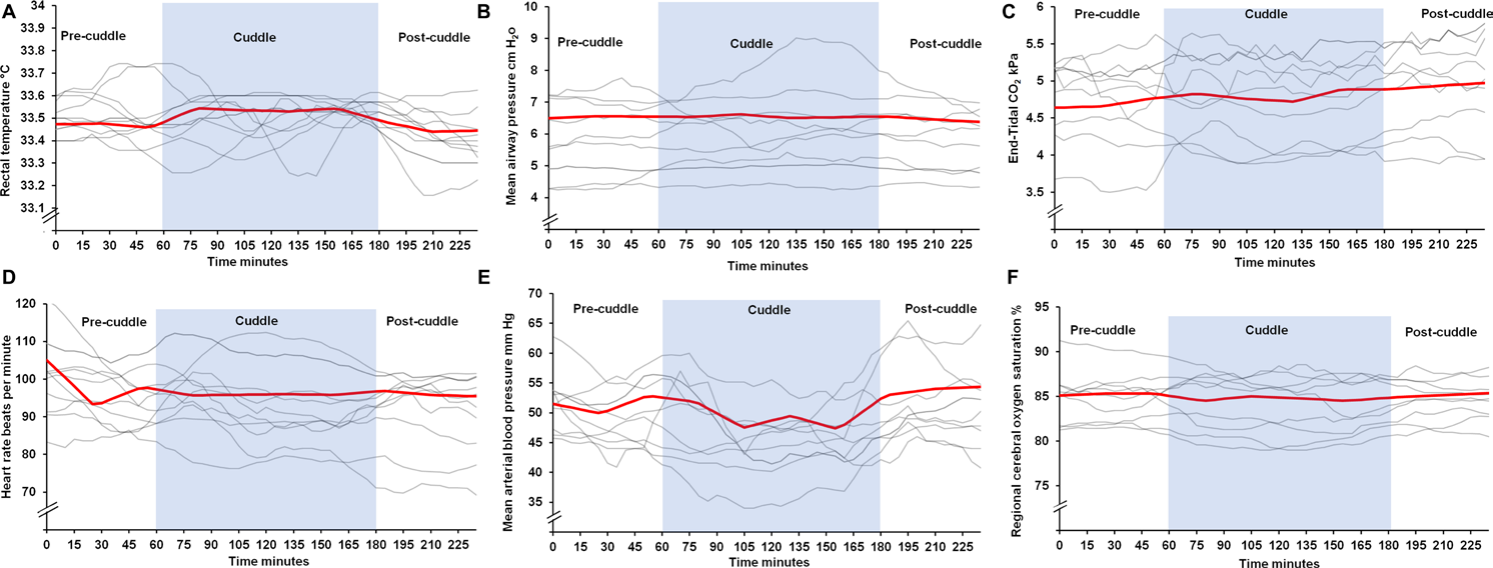
Change in primary outcome variables including rectal temperature, mean airway pressure, end-tidal CO_2_, heart rate, mean arterial blood pressure and regional cerebral oxygen saturation during CoolCuddle process. Trend line (red) showing linear regression with 30 min spline-points (derived from all 2-hour cuddle data). Additional plots are data of first 10 infants with complete data as examples of individual patient variation and trajectories. Shaded area shows period of CoolCuddle. The mean arterial blood pressure in the lowest line in figure E did not remain consistently 10 mm Hg below the precuddle level for greater than 20 min to stop the cuddle.

There was no evidence of a clinically meaningful difference in most measures between the precuddle, during and postcuddle periods (table 2), although peripheral (p=0.0048) and rectal temperature (p=0.0006) varied during the study; with increased rectal temperature during the cuddle (0.07°C (0.03–0.10)) (table 3)(online supplemental figure 2). In addition, there were changes in peripheral oxygen saturation % (p=0.0213) and end-tidal carbon dioxide kPa (p<0.001) (tables 2 and 3, figure 2). There was no difference in peak inspiratory pressure, peak end expiratory pressure, mean airway pressure, fraction of inspired oxygen, inspiratory time, tidal volume or respiratory rate between the three observation periods. During two cuddles, blood gas analysis was performed, which showed higher levels of partial pressures of carbon dioxide afterwards (0.44 kPa (0.22–0.66)), but no differences in pH, partial pressures of oxygen, base deficit, glucose or lactate between the three observation periods. There were changes in mean BP (p=0.0287), with babies having higher mean BP after the cuddle (4.09 mm Hg (0.96–7.21)), but no difference in the measures of heart rate or regional cerebral oxygenation. Finally, while occurrence of seizures and sleep–wake cycling, and the overall aEEG score did not vary between the three periods; the aEEG upper margin (p<0.001) and bandwidth (p<0.001) did change during the cuddle. Pain measures were similar throughout (p=0.98) and analgesia dose was not changed during any cuddles. No infant met the criteria to stop the cuddles (online supplemental table 4) or had any predefined adverse events. Analysis of the cuddles performed during rewarming are presented in online supplemental tables 5 and 6.

10.1136/bmjpo-2021-001280.supp2 Supplementary data

**Table 2:** Summary values of the respiratory, cardiovascular haemodynamics and core temperature data (CoolCuddles during cooling)

Variable	N*	Precuddle	N*	During cuddle	N*	Postcuddle	P value†
Respiratory parameters							
PIP (cmH_2_0)	54	12.6 (5.1)	54	13.4 (5.1)	53	12.2 (5.1)	0.0819
PEEP (cmH_2_0)	54	5.0 (0.7)	54	5.1 (0.7)	53	5.0 (0.6)	0.1576
MAP (cmH_2_0)	54	6.5 (1.9)	54	6.6 (1.8)	53	6.3 (1.5)	0.4165
FiO_2_ (%)	58	21.1% (21.0%–21.2%)	58	21.2% (21.0%–21.4%)	57	21.2% (20.9%–21.3%)	0.5765
SaO_2_ (%)	58	98.8% (98.4%–99.2%)	58	98.2 (97.6%–98.9%)	57	99.0% (98.4%–99.5%)	0.0213
T_I_ (s)	54	0.43 (0.04)	54	0.43 (0.04)	53	0.43 (0.04)	0.4602
ET-CO_2_ (kPa)	48	4.7 (0.7)	48	4.8 (0.7)	47	5.0 (0.7)	<0.001
Tidal volume (mL)	54	18.7 (4.3)	54	17.7 (3.3)	53	18.3 (3.6)	0.0615
Respiratory rate	58	34.0 (8.7)	58	34.0 (8.1)	57	35.0 (9.2)	0.1765
Blood gas measures							
pH	24	7.37 (0.05)	2	7.37 (0.01)	23	7.36 (0.4)	0.1542
PO_2_ (kPa)	24	8.7 (4.9)	2	6.3 (1.0)	23	7.5 (3.9)	0.1048
PCO_2_ (kPa)	24	5.3 (1.0)	2	4.7 (0.6)	23	5.5 (0.8)	0.0004
Base deficit (mEq/L)	24	−2.7 (3.2)	2	−4.7 (1.3)	23	−3.0 (2.0)	0.9661
Glucose (mmol/L)	24	4.9 (4.5–5.3)	2	4.3 (0.6–29.1)	23	4.4 (3.9–4.9)	0.5802
Lactate (mmol/L)	24	1.3 (0.9–1.7)	2	1.2 (0.4–3.5)	23	1.2 (0.9–1.6)	0.8190
Cardiovascular							
Mean BP (mm Hg)	58	48.5 (6.7)	58	49.5 (14.7)	57	52.6 (8.7)	0.0287
Heart rate (beats/min)	58	97.9 (25.3)	58	96.2 (14.6)	57	95.7 (13.4)	0.7200
Neurology							
rSCo_2_(%)	54	84.5 (4.0)	55	84.4 (5.4)	54	84.5 (3.9)	0.9768
Seizures	57	1 (1.8%)	58	1 (1.7%)	56	1 (1.8%)	>0.999
Abnormal aEEG‡	57	20 (35.1%)	58	26 (44.8%)	56	20 (35.7%)	0.2105
aEEG							
Lower margin voltage (µV)	55	6.1 (2.3)	55	5.9 (2.1)	53	6.1 (2.1)	0.6609
Upper margin voltage (µV)	55	17.2 (5.7)	55	19 (5.4)	53	17.2 (5.2)	<0.001
Bandwidth (µV)	55	11.1 (5.2)	55	13.1 (4.7)	53	11.1 (4.3)	<0.001
Sleep–wake cycling	58	25 (43.1%)	57	25 (43.9%)	55	24 (43.6%)	0.9619
Pain score >0	56	21 (37.5%)	58	21 (36.2%)	56	21 (37.5%)	0.9791
Temperature							
Peripheral temp (°C)	58	30.47 (1.14)	58	30.70 (0.93)	57	30.30 (1.24)	0.0048
Rectal temp (°C)	58	33.47 (0.11)	58	33.54 (0.09)	57	33.47 (0.15)	0.0006

Values are arithmetic mean (SD), geometric mean (95% CI) or number (%) as appropriate.

Seizures were not of status epilepticus nature to prevent recruitment for CoolCuddle.

*Number of cuddles for which data were available.

†P value from multilevel model accounting for dependent data for infants and cuddles.

‡aEEG pattern of discontinuous voltage, burst suppression, low voltage or flat trace.

aEEG, amplitude-integrated electroencephalogram; BP, blood pressure; ET-CO_2_, end-tidal carbon dioxide; FiO_2_, fraction of inspired oxygen; MAP, mean airway pressure; PCO_2_, partial pressures of carbon dioxide; PEEP, peak end expiratory pressure; PIP, peak inspiratory pressure; PO_2_, partial pressures of oxygen; rScO_2_, regional cerebral oxygenation; SaO_2_, peripheral oxygen saturation; T_I_, inspiratory time.

**Table 3:** Changes in summary values of the respiratory, cardiovascular haemodynamics and core temperature data compared with precuddle period (primary cohort, just cooled)

Variable	N*	Precuddle	N*	During cuddle	N*	Postcuddle
Respiratory parameters						
PIP (cmH_2_0)	54	Ref	54	0.80 (−0.11 to 1.70)	53	−0.19 (−1.10 to 0.72)
PEEP (cmH_2_0)	54	Ref	54	0.07 (−0.01 to 0.15)	53	0.01 (−0.07 to 0.09)
MAP (cmH_2_0)	54	Ref	54	0.05 (−0.13 to 0.22)	53	−0.07 (−0.25 to 0.10)
FiO_2_ (%)	58	Ref	58	0.00 (−0.00 to 0.00)	57	0.00 (−0.00 to 0.00)
SaO_2_ (%)	58	Ref	58	−0.57 (−1.08 to −0.05)	57	0.12 (−0.40 to 0.63)
T_I_ (s)	54	Ref	54	−0.00 (−0.00 to 0.00)	53	−0.00 (−0.00 to 0.00)
ET-CO_2_ (kPa)	48	Ref	48	0.09 (−0.01 to 0.19)	47	0.25 (0.14 to 0.35)
Tidal volume (mL)	54	Ref	54	−1.00 (−2.03 to 0.04)	53	0.16 (−0.88 to 1.20)
Respiratory rate	58	Ref	58	−0.07 (−1.69 to 1.56)	57	1.31 (−0.32 to 2.95)
Blood gas measures						
pH	24	Ref	2	−0.03 (−0.08 to 0.02)	23	−0.01 (−0.03 to 0.00)
PO_2_ (kPa)	24	Ref	2	−3.46 (−7.83 to 0.91)	23	−1.42 (−3.00 to 0.15)
PCO_2_ (kPa)	24	Ref	2	0.45 (−0.22 to 1.12)	23	0.44 (0.22 to 0.66)
Base deficit (mEq/L)	24	Ref	2	−0.27 (−2.60 to 2.06)	23	0.03 (−0.79 to 0.85)
Glucose (mmol/L)	24	Ref	2	−0.36 (−1.78 to 1.06)	23	−0.27 (−0.79 to 0.26)
Lactate (mmol/L)	24	Ref	2	−0.26 (−1.54 to 1.02)	23	−0.13 (−0.59 to 0.32)
Cardiovascular						
Mean BP (mm Hg)	58	Ref	58	1.00 (−2.11 to 4.12)	57	4.09 (0.96 to 7.21)
Heart rate (bpm)	58	Ref	58	−1.66 (−5.78 to 2.46)	57	−1.17 (−5.31 to 2.98)
SPO_2_ (%)	51	Ref	52	−0.82 (−1.40 to 0.25)	51	−0.34 (−0.92 to 0.23)
Neurology						
rSCo_2_ (%)	54	Ref	55	−0.08 (−1.11 to 0.94)	54	−0.11 (−1.14 to 0.92)
Seizures (OR)	57	Ref	58	0.99 (0.02 to 55.29)	56	1.01 (0.02 to 56.43)
Abnormal aEEG† (OR)	57	Ref	57	2.58 (0.76 to 8.76)	56	1.05 (0.32 to 3.47)
aEEG measures						
Lower margin voltage (µV)	55	Ref	55	−0.17 (−0.63 to 0.29)	53	0.03 (−0.44 to 0.49)
Upper margin voltage (µV)	55	Ref	55	1.80 (0.83 to 2.72)	53	−0.41 (−1.3 to 0.55)
Bandwidth (µV)	55	Ref	55	1.95 (0.90 to 3.01)	53	−0.41 (−1.48 to 0.66)
Sleep–wake cycling (OR)	55	Ref	55	1.29 (0.19 to 8.86)	53	1.08 (0.15 to 7.55)
Pain score >0	56	Ref	58	0.91 (0.32 to −2.58)	56	1.00 (0.35 to −2.84)
Temperature						
Peripheral temp (°C)	58	Ref	58	0.23 (−0.01 to 0.47)	57	−0.17 (−0.41 to 0.07)
Rectal temp (°C)	58	Ref	58	0.07 (0.03 to 0.10)	57	−0.00 (−0.04 to 0.04)

Values are SD of the mean (95% CI) or OR (95% CI) as appropriate from the precuddle period.

*Number of cuddles for which data were available.

†aEEG pattern of discontinuous voltage, burst suppression, low voltage or flat trace.

aEEG, amplitude-integrated electroencephalogram; BP, blood pressure; ET-CO_2_, end-tidal carbon dioxide; FiO_2_, fraction of inspired oxygen; MAP, mean airway pressure; PCO_2_, partial pressures of carbon dioxide; PEEP, peak end expiratory pressure; PIP, peak inspiratory pressure; PO_2_, partial pressures of oxygen; rScO_2_, regional cerebral oxygenation; SaO_2_, peripheral oxygen saturation; T_I_, inspiratory time.

After testing to see if the associations and profiles of the six main outcomes variables varied by the cooling period (cooled vs rewarmed), the number of the cuddle (eg, first, second, etc.), the parent cuddling and the grade of HIE), we found evidence that in rewarming cuddles, the stability of the rectal temperature did vary (p<0.001), but there was no evidence that the profile of measures differed during rewarming and no evidence that any measure varied by parent cuddling, HIE grade or number of cuddle (all p>0.0.10).

Between 5–7 days and 8 weeks postpartum, the mother–infant bonding scores were similar, EPDS scores decreased with a similar reduction in number of mothers with depression. Mothers reported breastfeeding in 71% and 50% of infants at 5–7 days and 8 weeks postpartum, which was higher than national average (46.5%–54.6%) at discharge for cooled babies.^21^ Median score of paternal postnatal attachment score at 8 weeks postpartum was 77 (71–83). (online supplemental table 7).

## Discussion

In this study, we developed a SOP for enabling parents to cuddle their infants during TH and intensive care across two NICUs. We were able to identify measurable changes in core and peripheral temperature, peripheral oxygen saturation and voltage of upper margin of aEEG during the cuddle and changes in the end-tidal CO_2_, and mean BP postcuddle. However, these changes were not clinically significant, and none of the infants reached the predefined thresholds for stopping the cuddle or experienced adverse effects. The work was not specifically powered to detect differences between any individual physiological measures and so the lack of statistical significance in some of the measures should be interpreted with caution. Between 5–7 days and 8 weeks postpartum, maternal postnatal depression scores decreased and mother to infant bonding scores remained stable. About 70% of infants received breast milk before discharge. Paternal postnatal attachment scores at 8 weeks postpartum were similar to published norms.

When TH is interrupted by the cuddling process, we would anticipate a rise in surface temperature of the baby and changes in the heart rate, BP, peripheral oxygen saturation and potentially ventilation due to cuddling and moving the infant from the cot. All infants received servo-controlled cooling therapy using a cooling wrap covering most of the infant’s body surface except the extremities, where the skin-to-skin contact occurred between the parents and their infants. This appears to have increased the peripheral temperature by a mean of 0.23°C and consequently core temperature by 0.07°C during the cuddle, which is clinically insignificant. Therefore, using insulating foam between the parent and infant while cuddling during TH^22^ is unnecessary.

Peripheral oxygen saturation decreased during cuddles and ET CO_2_, PCO_2_ and mean BP rose after the cuddle compared with precuddle. Skin-to-skin contact in preterm infants has been reported to have no effect^23^ or decrease the respiratory rate and peripheral oxygen saturation.^24^ Furthermore, skin-to-skin contact in a prone position on a parent chest seated on a reclining chair at 30° was reported to favour ventilation of the dorsal lung more than ventral lung.^25^ It appears that cuddling combined with the cooling wrap might restrict the chest excursion limiting the tidal volume as seen during cuddle (table 2), and the unaltered respiratory rate or the position of the infant during a cuddle might contribute to changes in ventilation. Despite these physiological changes, peripheral oxygen saturation and CO_2_ were within clinically acceptable levels and no infant breached the predefined thresholds consistently to stop the cuddle. Given that there was no change in the heart rate, elevated mean BP may be due to high systemic vascular resistance induced by peripheral vasoconstriction caused by lowering of skin temperature after the cuddle^26^ to achieve the target core temperature. While cerebral autoregulation may be impaired in acute HIE,^27^ the CoolCuddle was not associated with changes in regional cerebral oxygenation, a marker of cerebral blood flow. It was similar to the stable regional cerebral oxygenation reported in preterm infants having skin-to-skin contact while receiving respiratory support, although these infants had stable temperatures and their BP was not monitored.^28^ This suggests that parents cuddling infants with severe encephalopathy during cooling might not affect the cerebral blood flow.

We noted that during the cuddle, the voltage of upper margin of aEEG increased, suggesting high amplitude electrical activity. We used consistent assessment techniques to measure the voltage of upper and lower margins of the aEEG to limit any potential bias. Frontal alpha asymmetry assessed using 128 channel EEG representing emotional regulatory process during mother–infant interaction was higher in dyads with more responsive than less responsive mothers.^29^ We used one channel EEG and whether the high amplitude electrical activity seen during cuddle represents emotional regulatory process between parents and infants is not known.

As expected, EPDS scores at 5–7 days were higher than at 8 weeks postpartum, but the scores were also higher (worse) than those in population studies^30 31^ and in a US study of mothers of infants with HIE.^32^ Breastfeeding is negatively associated with postnatal depression,^33^ but breastfeeding rates at discharge and 8 weeks in this study were higher than those seen in babies undergoing standard care nationally.^21^

Given that parents cuddling babies receiving intensive care is standard of care in the NICU and the lack of clinically significant impact on cooling therapy and intensive care with CoolCuddle, our discussions with parents of cooled infants and clinicians participating in the study indicated that parents would decline to be randomised into non-cuddle arm, and it may not be practical or acceptable to randomise parents between ‘CoolCuddle’ and ‘no-CoolCuddle’ arms. While we acknowledge that a randomised controlled trail might offer definitive evidence regarding the efficacy of CoolCuddle, it may not be feasible to undertake such a trial. Therefore, we propose to roll out the CoolCuddle in few NICUs evaluating the process of embedding CoolCuddle in routine practice and monitoring the impact of CoolCuddle on cooling therapy, intensive care, parental mood and bonding.

Strengths of this study include involving two sites with different clinical teams, and both parents, as well as unwell ventilated infants and those receiving cardiovascular support. Limitations of this study include a lack of a comparison group to assess the impact of cuddling infants on parent–infant bonding or attachment and postnatal depression scales. While the infants acted as their own control for assessing the effect of cuddling on physiology, it was not feasible to obtain bonding scores and postnatal depression scores prior to administering the cuddles, to assess the immediate effects of cuddling. In addition, delays in the transfer of the mothers, sometimes many hours after their infants, to the centre offering cooling therapy, and the need for consent in this work inevitably led to the first cuddle taking place after their first day of life. Finally, CoolCuddle was overseen by an experienced nurse practitioner and two to three nurses, and it remains to be seen whether the intervention can be safely implemented in other NICUs with different working practices and intensive care environments. The work was also not specifically powered to detect differences between any individual physiological measures. While for many of the measures, the estimates for any real change were quite precise (eg, the 95% CI for heart rate was likely to vary by between −2 and 4 beats per minute in cuddle period compared with the precuddle period), for others, the precision was low and uninterpretable (eg, the OR of seizures had a CI of 0.02 to 55.29 for the cuddle period compared with the precuddle period) although a-priori safety ranges were not exceeded during any of the cuddles.

## Conclusion

In this work, we were able to identify small effects on cardiorespiratory physiology and brain activity as parents cuddled their infants receiving cooling therapy and intensive care for HIE. Maternal postnatal depression scores declined from 1 to 8 weeks postpartum and parent–infant bonding was stable. Seventy per cent of infants received breast milk at discharge and 50% breastfed at 8 weeks. While meticulous observation and administration of cuddles using a SOP in this study did not lead to any clinically relevant changes, the CoolCuddle intervention requires investigation in other NICU settings with a larger sample before widespread implementation.

## Data Availability

Data are available upon reasonable request. All data relevant to the study are included in the article or uploaded as supplementary information. Data for supplementary material will be available as deidentified individual participant data following necessary regulatory approvals.

## References

[1] NICE. Therapeutic hypothermia with intracorporeal temperature monitoring for hypoxic perinatal brain injury: NICEIPG347, 2010. Available: http://www.nice.org.uk/nicemedia/live/11315/48809/48809.pdf

[2] Shankaran S, Pappas A, McDonald SA, et al. Childhood outcomes after hypothermia for neonatal encephalopathy. N Engl J Med 2012;366:2085–92. 10.1056/NEJMoa1112066 22646631 PMC3459579

[3] Azzopardi D, Strohm B, Marlow N, et al. Effects of hypothermia for perinatal asphyxia on childhood outcomes. N Engl J Med 2014;371:140–9. 10.1056/NEJMoa1315788 25006720

[4] Lee-Kelland R, Jary S, Tonks J, et al. School-Age outcomes of children without cerebral palsy cooled for neonatal hypoxic-ischaemic encephalopathy in 2008-2010. Arch Dis Child Fetal Neonatal Ed 2020;105:8–13. 10.1136/archdischild-2018-316509 31036702

[5] Spencer APC, Brooks JCW, Masuda N, et al. Disrupted brain connectivity in children treated with therapeutic hypothermia for neonatal encephalopathy. Neuroimage Clin 2021;30:102582. 10.1016/j.nicl.2021.102582 33636541 PMC7906894

[6] de Cock ESA, Henrichs J, Klimstra TA, et al. Longitudinal associations between parental bonding, parenting stress, and executive functioning in Toddlerhood. J Child Fam Stud 2017;26:1723–33. 10.1007/s10826-017-0679-7 28572718 PMC5429904

[7] Thyagarajan B, Baral V, Gunda R, et al. Parental perceptions of hypothermia treatment for neonatal hypoxic-ischaemic encephalopathy. J Matern Fetal Neonatal Med 2018;31:2527–33. 10.1080/14767058.2017.1346074 28637367

[8] Bäcke P, Hjelte B, Hellström Westas L, et al. When all I wanted was to hold my baby-The experiences of parents of infants who received therapeutic hypothermia. Acta Paediatr 2021;110:480–6. 10.1111/apa.15431 32564441

[9] Heringhaus A, Blom MD, Wigert H. Becoming a parent to a child with birth asphyxia-From a traumatic delivery to living with the experience at home. Int J Qual Stud Health Well-being 2013;8:20539–13. 10.3402/qhw.v8i0.20539 PMC3643077 23639330

[10] Horsch A, Jacobs I, Gilbert L, et al. Impact of perinatal asphyxia on parental mental health and bonding with the infant: a questionnaire survey of Swiss parents. BMJ Paediatr Open 2017;1:e000059. 10.1136/bmjpo-2017-000059 PMC5862159 29637108

[11] Jordan E, Grant M, Bhakthavalsala S. Survey of sedation, respiratory support and parental contact practices during therapeutic hypothermia for hypoxic-ischaemic encephalopathy. Dublin: Neonatal Society, 2018: 21.

[12] Hypothermia as a neuroprotective intervention for neonatal encephalopathy-Bristol cooling therapy pathway 2015.

[13] Medicine BAoP. Therapeutic hypothermia for neonatal encephalopathy a BAPM framework for practice: British Assocation of perinatal medicine, 2020. Available: https://www.bapm.org/resources/237-therapeutic-hypothermia-for-neonatal-encephalopathy

[14] Chakkarapani E, Beasant L, Okano S. CoolCuddle study protocol and statistics analysis plan, 2021. Available: 10.5523/bris.3vs3y2wa9t4if2jo97ni4x4cae

[15] Gundersen JK, Chakkarapani E, Jary S, et al. Morphine and fentanyl exposure during therapeutic hypothermia does not impair neurodevelopment. EClinicalMedicine 2021;36:100892. 10.1016/j.eclinm.2021.100892 34308308 PMC8257990

[16] Hummel P, Lawlor-Klean P, Weiss MG. Validity and reliability of the N-PASS assessment tool with acute pain. J Perinatol 2010;30:474–8. 10.1038/jp.2009.185 19924132

[17] Cox JL, Holden JM, Sagovsky R. Detection of postnatal depression. development of the 10-item Edinburgh postnatal depression scale. Br J Psychiatry 1987;150:782–6. 10.1192/bjp.150.6.782 3651732

[18] Taylor A, Atkins R, Kumar R, et al. A new mother-to-infant bonding scale: links with early maternal mood. Arch Womens Ment Health 2005;8:45–51. 10.1007/s00737-005-0074-z 15868385

[19] Condon JT, Corkindale CJ, Boyce P. Assessment of postnatal paternal–infant attachment: development of a questionnaire instrument. J Reprod Infant Psychol 2008;26:195–210. 10.1080/02646830701691335

[20] Levis B, Negeri Z, Sun Y, et al. Accuracy of the Edinburgh postnatal depression scale (EPDS) for screening to detect major depression among pregnant and postpartum women: systematic review and meta-analysis of individual participant data. BMJ 2020;371:m4022. 10.1136/bmj.m4022 33177069 PMC7656313

[21] Gale C, Longford NT, Jeyakumaran D, et al. Feeding during neonatal therapeutic hypothermia, assessed using routinely collected national neonatal research database data: a retrospective, UK population-based cohort study. Lancet Child Adolesc Health 2021;5:408–16. 10.1016/S2352-4642(21)00026-2 33891879 PMC8131202

[22] Craig A, Deerwester K, Fox L, et al. Maternal holding during therapeutic hypothermia for infants with neonatal encephalopathy is feasible. Acta Paediatr 2019;108:1597–602. 10.1111/apa.14743 30721531 PMC6682469

[23] Heimann K, Vaessen P, Peschgens T, et al. Impact of skin to skin care, prone and supine positioning on cardiorespiratory parameters and thermoregulation in premature infants. Neonatology 2010;97:311–7. 10.1159/000255163 19887862

[24] Bohnhorst B, Heyne T, Peter CS, et al. Skin-to-skin (kangaroo) care, respiratory control, and thermoregulation. J Pediatr 2001;138:193–7. 10.1067/mpd.2001.110978 11174616

[25] Schinckel NF, Hickey L, Perkins EJ, et al. Skin-to-skin care alters regional ventilation in stable neonates. Arch Dis Child Fetal Neonatal Ed 2021;106:76–80. 10.1136/archdischild-2020-319136 32732379

[26] Thoresen M, Whitelaw A. Cardiovascular changes during mild therapeutic hypothermia and rewarming in infants with hypoxic-ischemic encephalopathy. Pediatrics 2000;106:92–9. 10.1542/peds.106.1.92 10878155

[27] Chakkarapani E, Dingley J, Aquilina K, et al. Effects of xenon and hypothermia on cerebrovascular pressure reactivity in newborn global hypoxic-ischemic pig model. J Cereb Blood Flow Metab 2013;33:1752–60. 10.1038/jcbfm.2013.123 23899927 PMC3824173

[28] Lorenz L, Dawson JA, Jones H, et al. Skin-to-skin care in preterm infants receiving respiratory support does not lead to physiological instability. Arch Dis Child Fetal Neonatal Ed 2017;102:F339–44. 10.1136/archdischild-2016-311752 28096239

[29] Perone S, Gartstein MA, Anderson AJ. Dynamics of frontal alpha asymmetry in mother-infant dyads: insights from the still face paradigm. Infant Behav Dev 2020;61:101500. 10.1016/j.infbeh.2020.101500 33197784

[30] Vigod SN, Villegas L, Dennis C-L, et al. Prevalence and risk factors for postpartum depression among women with preterm and low-birth-weight infants: a systematic review. BJOG 2010;117:540–50. 10.1111/j.1471-0528.2009.02493.x 20121831

[31] Paul E, Pearson RM. Depressive symptoms measured using the Edinburgh postnatal depression scale in mothers and partners in the ALSPAC study: a data note. Wellcome Open Res 2020;5:108. 10.12688/wellcomeopenres.15925.1 32766456 PMC7385546

[32] Laudi A, Peeples E. The relationship between neonatal encephalopathy and maternal postpartum depression. J Matern Fetal Neonatal Med 2020;33:3313–7. 10.1080/14767058.2019.1571574 30651011

[33] Demontigny F, Girard M-E, Lacharité C, et al. Psychosocial factors associated with paternal postnatal depression. J Affect Disord 2013;150:44–9. 10.1016/j.jad.2013.01.048 23489392

