# Supplementary material for: Physiological responses to cuddling babies with hypoxic–ischaemic encephalopathy during therapeutic hypothermia: an observational study
Source: BMJ Paediatr Open. 2021 Dec 16;5(1):e001280. doi: 10.1136/bmjpo-2021-001280 (PMC8679081; doi:10.1136/bmjpo-2021-001280)
Supplement: Supplementary data [file bmjpo-2021-001280supp002.pdf]

SUPPLEMENTARY MATERIAL

Impact of parents cuddling babies during therapeutic hypothermia and intensive care: a prospective observational intervention assessment study.

David Odd MD[1], Satomi Okano[2], Jenny Ingram PhD[3], Pete S Blair PhD[4], Amiel Billetop MRCPCH[5] , Peter Fleming PhD[4], Marianne Thoresen PhD[6,7], Ela Chakkarapani MD[2,6].

[1]Division of Population Medicine, Neuadd Meirionnydd, University Hospital of Wales, Heath Park, Cardiff, CF14 4YS

[2]Regional Neonatal Intensive Care Unit, St Michael's Hospital, University Hospitals Bristol and Weston NHS Foundation Trust. Southwell street, Bristol BS2 8EG.

[3]Centre for Academic Child Health, Bristol Medical School, University of Bristol, 1-5 Whiteladies Rd, Bristol BS8 1NU.

[4]Centre for Academic Child Health, Bristol Medical School, University of Bristol,
St Michael's hospital, Southwell St, Bristol BS2 8EG

[5]Neonatal Intensive Care Unit, North Bristol NHS Trust, Bristol, BS10 5NB.

[6]Translational Health Sciences, Bristol Medical School, University of Bristol, Level D, Neonatal Neuroscience, St Michael's Hospital. Southwell St, Bristol BS2 8EG.

[7] Division of Physiology, Institute of Basic Medical Sciences, University of Oslo, Norway.

COOL CUDDLE

CUDDLING COOLED BABIES

COOL CUDDLE INTERVENTION

Funded by NIHR

NHS University Hospitals Bristol and Weston NHS Foundation Trust

1 Parent (Mum or Dad) sits in a chair with pillows on their lap.

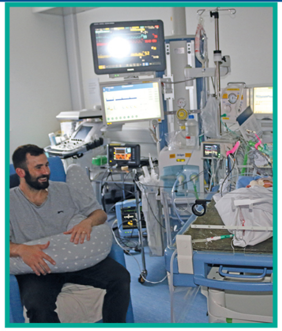

2 The wires and tubes around the baby are gathered into 2 bundles and secured with Velcro at either side of the baby.

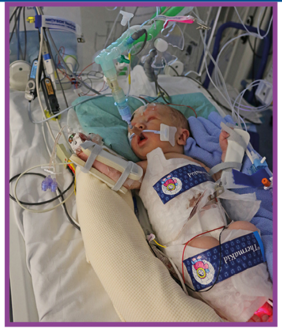

3 Baby (with wires) is wrapped in a sheet to keep everything secure.

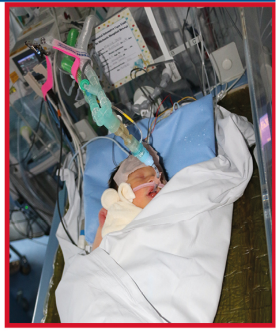

4 Two (or three) nurses carefully move the baby on to the pillow on the parent's lap.

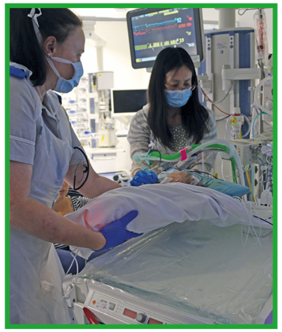

5 Cuddles can last for up to 2 hours.

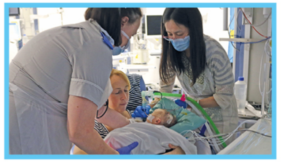

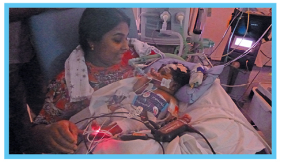

6 At the end of the cuddle the baby is moved back to the cot and made comfortable again.

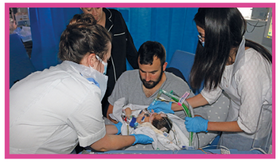

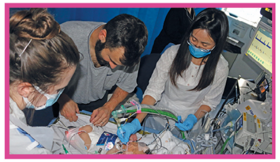

Online Supplementary Material

1. Supplementary Methods

OSM Table 1. Respiratory and Neurophysiology data collected during the study

**Category of data**	**Data collected**
Ventilation data (Drager VN500, Draeger Medical UK Ltd, Hemel Hempstead, UK)<br><br>End-tidal CO_2 (Philips Intellivue patient monitors, Philips, UK)	Peak inspiratory pressure (PIP), peak end expiratory pressure (PEEP), mean airway pressure (MAP), inspiratory time (T_I), tidal volume, respiratory rate and end-tidal CO_2 (ET-CO_2), fraction of inspired oxygen (FiO_2)
Blood gases if performed during the CoolCuddles	pH, partial pressures of oxygen (PO_2) and CO_2 (PCO_2), base deficit, glucose and lactate.
Near infrared spectroscopy (SenSmart Model X-100, NONIN, Terumo UK Ltd, UK)	regional cerebral oxygenation ($rScO_2$)
amplitude integrated electroencephalogram (aEEG) (CFM 6000 and CFM Olympic Brainz monitor, Natus Nicolet UK Ltd)	***Pattern***<br>0: continuous normal voltage<br>1: continuous normal voltage + seizures<br>2: discontinuous voltage<br>3: burst suppression<br>4: low voltage<br>5: flat trace.[1]<br><br>***Presence of sleep wake cycling***<br>Cyclic variations in the EEG background suggestive of sleep wake cycling was scored offline for the entire epoch of pre, during and post cuddles.<br><br>***Voltage of upper and lower margin of aEEG***<br>These were scored at 30 minutes after commencing pre-cuddle, cuddle (during 1st and 2nd hour of cuddle) and post-cuddle using a line that corresponded to the upper and lower margin of the dense band of aEEG (compressed EEG at 6cm/hour) on the digital screen.[2]

OSM Table 2. Criteria for stopping CoolCuddles during cooling

**Measures**	**Duration**	**Criteria**
Temperature	occurring continuously for 5 to10 minutes during cooling without responding to any potential resolvable causes	• Rectal temperature < 30·0°C or >35·0°C
Cardiovascular		• Mean blood pressure < 30mmHg or > 75 mmHg<br>• Heart rate < 50 beats per minute<br>• Heart rate >180 beats per minute
Respiratory		• Oxygen saturation < 80%<br>• Fraction of inspired oxygen >70%
Neurophysiology		• Electroclinical or electrical status epilepticus
Temperature	Occurring greater than 20 minutes after any remediable causes are attended to	• Rectal temperature between 30·0 and 32·9°C or between 34·1 and 34·9°C
Cardiovascular		• Mean blood pressure 10 mmHg below or above the pre-cuddle period<br>• Heart rate <20 beats per minute from the pre-Cuddle period or >20 beats per minute from the pre-Cuddle period
Respiratory		• Oxygen Saturation 80-88%<br>• Increase in Fraction of inspired oxygen by 20% above the pre-CoolCuddle period
General		• Medical or nursing concern that the infant is not adequately supported

OSM Table 3. Characteristics of cuddle for the participants.

**Cuddle 1 age hours**	**Cuddled by Mum (0) or Dad (1)**	**Cuddle 1 duration hours**	**Cuddle 2 age hours**	**Cuddled by Mum (0) or Dad (1)**	**Cuddle 2 duration hours**	**Cuddle 3 age hours**	**Cuddled by Mum (0) or Dad (1)**	**Cuddle 3 duration hours**	**Cuddle 4 age hours**	**Cuddled by Mum (0) or Dad (1)**	**Cuddle 4 duration hours**
40.15	1	1	64.07	0	2.00	75.07	1	2.00			
54.98	0	2	64.73	1	2.00	77.98	0	2.00			
59.43	0	1									
50.07	0	1	65.15	1	1.17	71.73	0	1.33			
51.87	0	2	74.03	1	2.00	81.45	0	1.00			
46.00	0	1.08	52.17	1	1.17	65.75	0	1.58			
55.43	0	0.75									
54.93	0	1.75	75.02	1	1.08						
62.62	0	1.92	70.20	1	2.00						
65.02	0	2									
53.10	0	2									
55.45	0	2	63.12	1	2.00	77.28	0	0.41			
87.15	0	1.83	98.15	0	2.00						
26.45	0	2	44.78	0	2.00	67.78	0	2.00			
40.50	1	1.17	47.33	0	2.00	64.83	1	2.00	72.42	0	2.00
32.52	1	2	39.27	0	1.58	57.43	0	2.00	65.68	1	2.00
32.48	0	1.92	45.32	1	1.58						
76.42	0	2									
47.27	1	1.67	70.68	1	2.00						
41.22	1	2	65.47	0	2.00						

41.12	0	0.83	54.95	1	1.42	63.70	0	1.50	75.95	1	2.00
43.72	0	1.17	50.38	1	1.33	64.63	0	2.00	72.38	1	2.00
61.78	0	2	80.70	0	1.17						
15.70	0	0.83	35.87	0	2.00	59.12	0	2.00	80.20	0	1.92
41.77	0	1.17	54.60	1	1.00	63.02	0	1.25	78.68	0	1.00
54.97	0	1.67	70.63	1	2.00	79.47	0	2.00			
51.85	1	2	70.35	0	0.75	78.43	0	2.00			

Odd D, *et al. BMJ Paediatrics Open* 2022; 5:e001280. doi: 10.1136/bmjpo-2021-001280

OSM Table 4. Number of cuddles that were outside of target ranges before, during and after Cuddle (Primary cohort, just Cooled)

Variable	N*	Pre-cuddle	N*	During cuddle	N*	Post cuddle	P value**
Rectal Temperature							
Temperature outside 30-35 degrees	58	0 (0·0%)	58	0 (0·0%)	57	1 (1·8%)	>0·999***
Mean BP							
< 30mmHg or > 75 mmHg	58	2 (3·5%)	58	1 (1·7%)	57	4 (7·0%)	0·2133
Heart Rate							
<50 or >180 beats per minutes	58	2 (3·5%)	58	0 (0·0%)	57	1 (1·8%)	0·548***
Oxygen Saturations							
<80%	58	0 (0·0%)	58	3 (3·5%)	57	1 (1·8%)	0·548***
FiO_2							
> 70%	58	4 (6·9%)	58	3 (5·2%)	57	2 (3·5%)	0·4650
Clinical or electrical status epilepticus	58	0 (0·0%)	58	0 (0·0%)	57	0 (0·0%)	>0·999***

* number of cuddles, ** p value from multi-level model accounting for dependent data for infants and cuddles, or *** from simple frequency comparisons due to small numbers/limited comparisons. Babies who crossed the threshold during cuddle did not stay beyond the threshold consistently for 10 minutes. FiO_2 Fraction of inspired oxygen

OSM Table 5. Summary values of the respiratory, cardiovascular haemodynamics and core temperature data (Only re-warming Infants)

Variable	N*	Pre-cuddle	N*	During cuddle	N*	Post cuddle	P value**
**Respiratory Parameters**							
PIP cmH20	11	10·0 (2·3)	11	11·6 (3·2)	11	10·8 (3·8)	0·2049
PEEP cmH20	11	5·1 (1·1)	11	4·8 (0·4)	11	4·8 (0·4)	0·4310
MAP cmH20	11	5·9 (1·0)	11	5·9 (0·8)	11	5·8 (0·9)	0·6076
FiO_2 %	12	0·21 (0·21-0·21)	12	0·21 (0·21-0·22)	12	0·21 (0·1-0·22)	0·3393
SaO_2 %	12	98·8 (97·5-100·0)	12	97·6 (95·9-99·3)	12	98·1 (96·8-99·3)	0·1519
T_I seconds	11	0·41 (0·03)	11	0·41 (0·03)	12	0·41 (0·02)	0·3340
ET-CO_2 kPa	11	4·9 (0·6)	11	4·9 (0·5)	11	4·9 (0·6)	0·5941
Tidal Volume ml	11	18·0 (4·9)	11	16·7 (2·0)	11	16·3 (2·3)	0·1850
Respiratory Rate	12	36·5 (10·0)	12	37·1 (9·6)	12	34·7 (5·5)	0·3143
**Blood Gas Measures**							
pH	4	7·40 (0·05)	0	-	5	7·40 (0·05)	-
pO_2 kPa	4	8·55 (2·1)	0	-	5	9·88 (2·3)	-
pCO_2 kPa	4	5·0 (0·36)	0	-	5	4·92 (0·61)	-
Base Deficit	4	-1·65 (3·9)	0	-	5	-2·2 (1·8)	-
Glucose mmol/L	4	4·1 (3·2-5·3)	0	-	5	4·8 (3·3-6·9)	-
Lactate mmol/L	4	0·7 (0·5-0·9)	0	-	5	1·0 (0·7-1·4)	-
**Cardiovascular**							
Mean BP mmHg	12	53·1 (11·4)	12	49·9 (9·3)	12	50·3 (9·9)	0·1841
Heart Rate beats/min	12	103 (12)	12	103 (12)	12	104 (13)	0·9427
$rSCo_2$%	12	97·3 (1·9)	12	96·8 (1·7)	12	98·0 (1·2)	0·0158
Mean BP mmHg	12	85·6 (3·4)	12	86·4 (3·3)	12	85·7 (3·5)	0·4003
**Neurology**							
Seizures	12	0 (0·0%)	11	0 (0·0%)	12	0 (0·0%)	>0·999
Abnormal aEEG***	11	5 (45·5%)	11	4 (36·4%)	12	3 (25·0%)	0·4008
aEEG							
Lower margin voltage µV	11	6·3 (2·3)	11	5·3 (1·5)	11	5·5 (2·0)	0·0891
Upper margin voltage µV	11	20·1 (6·7)	11	19·2 (6·3)	11	18 (6·3)	0·3094
Bandwidth µV	11	13·8 (5·6)	11	13·9 (5·4)	11	12·5 (5·4)	0·6310
Sleep Wake cycling	12	10 (83·3%)	12	10 (83·3%)	12	10 (83·3%)	-
Pain score >0	12	3 (25·0%)	11	3 (27·3%	11	3 (25·0%)	0·9382
**Temperature**							
Peripheral Temp °C	12	31·12 (0·87)	12	32·29 (9·08)	12	31·20 (1·24)	0·8005
Rectal Temp °C	12	34·26 (0·62)	12	34·77 (0·83)	12	35·18 (0·81)	<0·001

Values are mean (SD), geometric mean (94% CI), or number (%) as appropriate
* number of cuddles for which data was available
** p value from multi-level model accounting for dependent data for infants and cuddles
*** aEEG pattern of discontinuous voltage, burst suppression, low voltage or flat trace

PIP: Peak Inspiratory pressure
PEEP:Peak End Expiratory Pressure
MAP: Mean Airway Pressure
FiO_2: Fraction of Inspired Oxygen
SaO_2: Peripheral oxygen saturation

T_I: inspiratory time
ET-CO_2: End-tidal Carbon dioxide
PO_2: partial pressures of oxygen
PCO_2: partial pressures of carbon dioxide
BP: Blood pressure
$rScO_2$: regional cerebral oxygenation
aEEG: amplitude integrated electroencephalogram

Odd D, *et al. BMJ Paediatrics Open* 2022; 5:e001280. doi: 10.1136/bmjpo-2021-001280

OSM Table 6. Changes in summary values of the respiratory, cardiovascular haemodynamics and core temperature data compared to pre-cuddle period (Only re-warming Infants)

Variable	N*	Pre-cuddle	N*	Cuddle	N*	Post cuddle
**Respiratory Parameters**						
PIP cmH_20	11	Ref	11	1·53 (-0·10 to 3·16)	11	0·72 (-0·91 to 2·35)
PEEP cmH_20	11	Ref	11	-0·28 (-0·73 to 0·18)	11	-0·25 (-0·71 to 0·20)
MAP cmH_20	11	Ref	11	-0·03 (-0·41 to 0·35)	11	-0·18 (-0·56 to 0·20)
FiO_2 %	12	Ref	12	0·00 (-0·00 to 0·01)	12	0·00 (-0·00 to 0·01)
SaO_2 %	12	Ref	12	-1·19 (-2·35 to 0·02)	12	-0·74 (-1·91 to 0·42)
T_I seconds	11	Ref	11	0·00 (-0·00 to 0·00)	12	0·00 (-0·00 to 0·00)
ET-CO_2 kPa	11	Ref	11	-0·04 (-0·20 to 0·12)	11	0·04 (-0·12 to 0·20)
Tidal Volume ml	11	Ref	11	-1·30 (-3·15 to 0·55)	11	-1·73 (-3·59 to 0·12)
Respiratory Rate	12	Ref	12	0·58 (-0·25 to 3·69)	12	-1·79 (-4·89 to 1·32)
**Blood Gas Measures**						
pH	4	Ref	0	-	5	0·01 (-0·05 to 0·06)
pO_2 kPa	4	Ref	0	-	5	1·33 (-2·19 to 4·85)
pCO_2 kPa	4	Ref	0	-	5	-0·08 (-0·91 to 0·75)
Base Deficit	4	Ref	0	-	5	-0·57 (-5·20 to 4·06)
Glucose mmol/L	4	Ref	0	-	5	0·77 (-1·05 to 2·58)
Lactate mmol/L	4	Ref	0	-	5	0·32 (-0·03 to 0·66)
**Cardiovascular**						
Mean BP mmHg	12	Ref	12	-3·15 (-6·71 to 0·41)	12	-2·80 (-6·36 to 0·75)
Heart Rate beats/min	12	Ref	12	-0·25 (-5·91 to 5·41)	12	0·71 (-4·95 to 6·36)
$rSCo_2$%	12	Ref	12	-0·51 (-1·24 to 0·22)	12	0·66 (-0·07 to 1·40)
Mean BP mmHg	12	Ref	12	0·74 (-0·44 to 1·92)	12	0·04 (-1·13 to 1·22)
**Neurology**						
Seizures (OR)	12	Ref	11	-	12	-
Abnormal aEEG** (OR)	11	Ref	11	0·44 (0·05 to 3·19)	12	0·23 (0·02 to 2·36)
aEEG Measures		Ref				
Lower margin voltage µV	11	Ref	11	-1·00 (-1·90 to -0·10)	11	-0·82 (-1·72 to 0·08)
Upper margin voltage µV	11	Ref	11	-0·91 (-3·52 to 1·71)	11	-2·09 (-4·71 to 0·52)
Bandwidth µV	11	Ref	11	0·09 (-2·99 to 3·17)	11	-1·27 (-4·36 to 1·81)
Sleep-Wake cycling (OR)	12	Ref	12	-	12	-
Pain score >0	12	Ref	11	1·42 (0·16-12·66)	11	1·00 (0·12-8·34)
**Temperature**						
Peripheral Temp°C	12	Ref	12	1·18 (-2·66 to 5·01)	12	0·08 (-3·76 to 3·92)
Rectal Temp°C	12	Ref	12	0·51 (0·32 to 0·70)	12	0·92 (0·73 to 1·11)

Values are mean increase (95% CI) or OR (95% CI) as appropriate
Some coefficients inestimable due to small numbers/limited comparisons
*number of cuddles for which data was available
** aEEG pattern of discontinuous voltage, burst suppression, low voltage or flat trace

PIP: Peak Inspiratory pressure
PEEP: Peak End Expiratory Pressure
MAP: Mean Airway Pressure
FiO_2: Fraction of Inspired Oxygen
SaO_2: Peripheral oxygen saturation
T_I: inspiratory time
ET-CO_2: End-tidal Carbon dioxide
PO_2: partial pressures of oxygen
PCO_2: partial pressures of carbon dioxide
BP: Blood pressure
$rScO_2$: regional cerebral oxygenation

Odd D, *et al. BMJ Paediatrics Open* 2022; 5:e001280. doi: 10.1136/bmjpo-2021-001280

aEEG: amplitude integrated electroencephalogram

OSM Table 7. Secondary outcomes

Variable	n	5-7 days	n	8 weeks	P value
Maternal-infant bonding scale	20	3 (0-6)	18	3 (1-4)	0·9175
Edinburgh postnatal depression Score	23		21		
Summary Score		13 (9-13)		9 (5-14)	0·0003
Score 13 or over		13 (56·5%)		5 (23·8%)	0·0078
Paternal postnatal attachment scale	15				
Summary Score		77 (71-83)			-
< 25th centile (74·8)		5 (40·0%)			0·180
< 10th centile (67·7)		1 (6·7%)			0·667
Feeding	24		24		
Breast milk		17 (71%)		12 (50%)	0.04
Mixed feeds		6 (25%)		4 (17%)	
Formula		1 (4%)		8 (33%)	

Numbers are median (IQR) or n(%) as appropriate

References

1. Hellström-Westas L, Rosén I, De Vries L, Greisen G. Amplitude-integrated EEG classification and interpretation in preterm and term infants. *NeoReviews* 2006; **7**(2): e76-e87.
2. al Naqeeb N, Edwards AD, Cowan FM, Azzopardi D. Assessment of neonatal encephalopathy by amplitude-integrated electroencephalography. *Pediatrics* 1999; **103**(6 Pt 1): 1263-71.
